# Diseases Neglected by the Media in Espírito Santo, Brazil in 2011–2012

**DOI:** 10.1371/journal.pntd.0004662

**Published:** 2016-04-26

**Authors:** Aline Guio Cavaca, Tatiana Breder Emerich, Paulo Roberto Vasconcellos-Silva, Edson Theodoro dos Santos-Neto, Adauto Emmerich Oliveira

**Affiliations:** 1 Post-graduate Program in Public Health, Federal University of Espírito Santo, Vitória, Espírito Santo, Brazil; 2 National School of Public Health Sérgio Arouca, Oswaldo Cruz Foundation, Rio de Janeiro, Brazil; The George Washington University School of Medicine and Health Sciences, UNITED STATES

## Abstract

**Background:**

The aims of the present study were to identify and analyse the Diseases Neglected by the Media (DNMs) via a comparison between the most important health issues to the population of Espírito Santo, Brazil, from the epidemiological perspective (health value) and their effective coverage by the print media, and to analyse the DNMs considering the perspective of key journalists involved in the dissemination of health topics in the state media.

**Methodology:**

Morbidity and mortality data were collected from official documents and from Health Information Systems. In parallel, the diseases reported in the two major newspapers of Espírito Santo in 2011–2012 were identified from 10,771 news articles. Concomitantly, eight interviews were conducted with reporters from the two newspapers to understand the journalists’ reasons for the coverage or neglect of certain health/disease topics.

**Principal Findings:**

Quantitatively, the DNMs identified diseases associated with poverty, including tuberculosis, leprosy, schistosomiasis, leishmaniasis, and trachoma. Apart from these, diseases with outbreaks in the period evaluated, including whooping cough and meningitis, some cancers, respiratory diseases, ischaemic heart disease, and stroke, were also seldom addressed by the media. In contrast, dengue fever, acquired immune deficiency syndrome (AIDS), diabetes, breast cancer, prostate cancer, tracheal cancer, and bronchial and lung cancers were broadly covered in the period analysed, corroborating the tradition of media disclosure of these diseases. Qualitatively, the DNMs included rare diseases, such as amyotrophic lateral sclerosis (ALS), leishmaniasis, Down syndrome, and verminoses. The reasons for the neglect of these topics by the media included the political and economic interests of the newspapers, their editorial line, and the organizational routine of the newsrooms.

**Conclusions:**

Media visibility acts as a strategy for legitimising priorities and contextualizing various realities. Therefore, we propose that the health problems identified should enter the public agenda and begin to be recognized as legitimate demands.

## Author Summary

The Diseases Neglected by the Media (DNMs) are those diseases without media visibility due to their low newsworthiness level. In most cases, these diseases affect deprived social groups. This study analyses the DNMs by comparing the space achieved by the most important health problems for the population of Espírito Santo, Brazil, in the local print media and the perceptions key journalists have of the media exposure in this social context. The major DNMs were poverty-related diseases such as tuberculosis, leprosy, schistosomiasis, leishmaniasis, trachoma and helminths. In addition, diseases with outbreaks during the analysed period, such as whooping cough and meningitis, along with some types of neoplasms, respiratory diseases, and ischaemic and cerebrovascular heart disease, were identified. It is believed that public authorities should count on a broader media space concerning diseases recognized as political issues. Therefore, the media visibility of health and political topics, as well as that of economics or any other subject, is strategic in regard to the legitimization of priorities and the contextualization of overlooked realities.

## Introduction

In the contemporary sociopolitical landscape, mass media operates in three dimensions, lending them a unique importance. It acts as a vehicle for the social dissemination of relevant information on health as a system that allows the introduction of social themes and issues to the public [1] and as an opinion-forming instrument for a significant portion of the population [2]. In addressing citizens, the media coverage of health issues should neither adopt persuasion as a strategy nor be limited to the goal of information dissemination. It should minimally promote public debate on topics of interest and—assuming that the right to information is inseparable from the right to health—should ensure the provision of information to increase public participation [1, 3] in building critical health awareness [2] and to stimulate social participation in the management of public health systems [4].

In this study, the media was evaluated considering its role of serving the public, which is essential to both attract commitments from new social actors, investments in research and the development of new drugs [5] and to disseminate current topics to the population. The aim of this study was to identify and to analyse the Diseases Neglected by the Media (DNMs) by classifying the most important health problems from an epidemiological perspective (health value) and their effective coverage by the print media in the state of Espírito Santo and from the perspective of key journalists involved in the media disclosure of health topics.

For this purpose, we assume that media visibility is a strategy used to legitimize priorities, contextualize realities, and organize and prioritize health problems on the public agenda, making these problems noticeable as concrete demands, specific and independent of the newsworthiness assigned to them by the current mode of news production. We believe in the political importance of the identification of DNMs, which are considered journalistically uninteresting because they do not meet the usual criteria of newsworthiness. These health problems and social conditions are neglected by the media but have an important “health value” (i.e., epidemiological relevance), proposed in this study as a relevant criterion in the process of determining publicity and coverage of public issues by assigning them different political weights and guiding unprecedented and urgent debates [6].

## Methods

This exploratory, quantitative, and qualitative study used a quantitative approach that allowed us to identify and classify and a qualitative approach that enabled us to understand, discuss, and interpret the topics evaluated. Therefore, these methodological approaches were complementary [7].

The study was divided into four phases:

### Phase I: Analysis of media coverage–selected sources

The coverages of the major newspapers of Espírito Santo—*A Gazeta* and *A Tribuna*—were evaluated for the period between January 1, 2011 and December 31, 2012.

At present, the structure of the media system in Espírito Santo and throughout Brazil is oligopolized. The daily newspaper *A Tribuna* has the largest circulation in the state, holding the eighteenth position in the Brazilian ranking of printed newspapers [8]. The newspaper *A Gazeta* is the oldest newspaper in circulation in the state, with 86 years of history, and is the leader in subscriptions in the state [9, 10].

#### Data collection

The archives of *A Tribuna* were provided free of charge, recorded on DVD, and periodically updated to cover the selected study period. PDF copies of *A Gazeta* were acquired from a clipping company. All of the news articles in these newspapers were evaluated by three trained researchers, who selected and classified health-related news.

#### Inclusion criteria

The study included the articles that referred to diseases as either the main focus (when the topic was the disease itself) or the secondary focus (when the main topic was another health-related topic but referred consistently to the disease).

#### Exclusion criteria

Articles that referred to diseases in a context irrelevant to the study, such as advertisements, promotion of private events, and public contest results, among others, were excluded. Similarly, external diseases and/or causes were excluded because they were not within the scope of the study.

#### Organization of the articles and evaluation of the frequency of disease reporting

The analysis records containing the characteristics of the definitions of the articles were completed on the basis of the articles selected. The diseases identified in the articles were classified according to the International Classification of Diseases (ICD-10), and the records were entered into an SPSS database (Version 18.0, SPSS Inc., Chicago, IL, USA).

A total of 10,771 health-related articles from the two newspapers were evaluated. In *A Tribuna*, 6,104 articles were selected, of which 60.4% referred to diseases. In *A Gazeta*, of the 4,667 articles analysed, 62.41% referred to diseases. Therefore, the present study analysed 6,601 newspaper articles about diseases, covering 10,435 diseases described in the ICD-10.

All of these diseases were classified according to their frequency of exposure in the study corpus. The most reported diseases in the print media of Espírito Santo in the study period were those that, together, represented 50% of all of the diseases cited in the examined articles (Table 1).

**Table 1:** Diseases Most Reported in the Newspapers *A Tribuna* and *A Gazeta* According to the International Classification of Diseases (ICD-10) in the State of Espírito Santo, Brazil, in 2011–2012.

Reported diseases (ICD-10)	N	%	Percentage accumulated
**Malignant neoplasms (C00-C97)**	718	6.9%	6.9%
**Diabetes (E10-E14)**	433	4.1%	11.0%
**Obesity (E66)**	381	3.7%	14.7%
**Dengue (A90)**	350	3.4%	18.0%
Stroke (I64)*	280	2.7%	20.7%
**Severe reactions to stress and adjustment disorders (F43)**	268	2.6%	23.3%
**Malignant breast neoplasms (C50)**	227	2.2%	25.5%
**Depression (F32-F33)**	224	2.1%	27.6%
**Myocardial infarction (I21-I22)**	214	2.1%	29.7%
**HIV (B20-B24)**	198	1.9%	31.6%
**Other muscle disorders (M62)**	182	1.7%	33.3%
**Hypertensive diseases (I10-I15)**	167	1.6%	34.9%
**Complications from heart disease and ill-defined heart disease (I51)**	167	1.6%	36.5%
**Alzheimer's disease (G30)**	144	1.4%	37.9%
**Malignant neoplasm of the larynx (C32)**	123	1.2%	39.1%
**Migraine (G43)**	123	1.2%	40.2%
**Influenza and pneumonia (J10-J18)**	119	1.1%	41.4%
**Rheumatic fever with heart involvement (I01)**	116	1.1%	42.5%
**Other anxiety disorders (F41)**	107	1.0%	43.5%
**Malignant neoplasm of the prostate (C61)**	100	1.0%	44.5%
**Circulatory diseases (excluding stroke) (I00-I99)**	100	1.0%	45.4%
**Viral hepatitis (B15-B19)**	95	0.9%	46.3%
**Disorders of lipoprotein metabolism and other lipidaemias (E78)**	80	0.8%	47.1%
**Malignant neoplasm of bronchi and lungs (C34)**	75	0.7%	47.8%
**Pains not classified elsewhere (R52)**	75	0.7%	48.5%
**Sleep disorders (G47)**	69	0.7%	49.2%
**Asthma (J45)**	68	0.7%	49.9%
**All other diseases**	5232	51.1%	100%

*Not specified as haemorrhagic or ischaemic.

### Phase II: Identification of the most epidemiologically important health problems

To identify the most epidemiologically important health problems, we analysed data on health policies and morbidity and mortality in Espírito Santo between 2011 and 2012 and developed a composite indicator designated “health value”.

This indicator represents a morbidity and mortality profile to indicate the health value of each disease for the media. This proposal was a point of approximation between the media relevance criteria (news value) and the health relevance criteria (health value) and was defined on the basis of four sources:

1. **Health policy priorities–**the 10 priority diseases reported in the 2012–2015 Espírito Santo State Health Plan [11] and the 2011 Master Plan for Regionalization [12] and the diseases identified as research priorities in the FAPES/CNPq/MS-Decit/SESA public call for research applications (no. 10/2013-PPSUS). These documents were selected as sources indicating the priorities in Espírito Santo because they are important instruments for health planning and the consequent establishment of policy priorities in the health sector.
2. **Major causes of death–**according to the Mortality Information System (Sistema de Informação de Mortalidade–SIM), which defined the top 10 causes of death in the state, following the ICD-10 criteria. These indicators are essential to identify the epidemiological profile of a certain geographical region, to analyse trends, recommend priorities, and evaluate programs.
3. **Major notifiable diseases**–defined as the top 10 diseases reported in the state according to the Information System for Notifiable Diseases (Sistema de Informação de Agravos de Notificação–SINAN). This system is the main source of information for epidemiological surveillance.
4. **Major causes of hospitalization in the Unified Health System–**defined as the top 10 causes of hospitalization in the state using data from the Hospital Information System of the Unified Health System (Sistema de Informações Hospitalares do Sistema Único de Saúde–SIH/SUS). These data are essential for the planning of healthcare costs. The ranking of diseases that composed this indicator followed the criterion of the decreasing frequency of mortality, morbidity, and notification. In the analysis of data on policy and research priorities, the criterion of coincidence of the disease in more than one policy selected was adopted, also in decreasing order of frequency [13].

The top 10 diseases in each category were grouped in Microsoft Office Excel 2010 and ranked according to the criterion of coincidence in two or more categories. This value indicated the health values of the most epidemiologically and politically important diseases in Espírito Santo.

### Phase III: Understanding media (non)coverage of health: The perceptions of key journalists

Eight semi-structured interviews were conducted with key journalists responsible for the health/disease agenda in the two major newspapers of Espírito Santo (*A Tribuna* and *A Gazeta*). These interviews were analysed using a content analysis technique [14].

The interviews were conducted individually and were based on a prepared script that included questions related to the reasons for the (non)coverage of topics on health/diseases in the print media.

The analysis technique used was thematic content analysis, which recommends the identification of the "core meaning" of the empirical material by the incorporation of several steps, including pre-analysis, exploration of the material (categorization), treatment of the results, inference, and interpretation (Bardin, 14).

The study was approved by the Research Ethics Committee of the Sérgio Arouca National School of Public Health and the Federal University of Espírito Santo. The newspaper agencies provided a formal authorization for the study to be conducted, and all interviewed participants signed an informed consent form.

### Phase IV: Identification of diseases neglected by the media: Quantitative and qualitative sources

A comparative matrix correlated the most relevant diseases in Espírito Santo considering epidemiological indicators (health value) with the corresponding media coverage. From there, DNMs were identified as conditions of high importance and limited media coverage. The diseases of high importance and high newsworthiness were also identified. These variables formed the media coverage indicators death-related news index, hospitalization-related news index, and notification-related news index, which represent the ratios of the number of news articles by the number of deaths, hospitalizations, and notifications, respectively, associated with morbid conditions in the study period. Therefore, rates much smaller than 1.0 tended to indicate low newsworthiness.

Concomitantly, the DNMs were identified, and the reasons for coverage or neglect by the media of topics on health/diseases in the opinion of the journalists interviewed were highlighted. These topics were discussed with respect to their agreement or disagreement with the quantitative results of the study.

## Results

The results indicate that the most covered diseases in Espírito Santo during the study period were malignant neoplasms (chapter C00-C97), followed by diabetes, obesity, dengue, stroke, and reactions to stress. Note that other malignancies, including breast, prostate, larynx, bronchial and lung cancers, appeared to be relevant when regional specifications were included (Table 1).

The morbid conditions ideally relevant for media coverage in Espírito Santo in 2011–2012 (health value) were defined on the basis of the state’s morbidity and mortality profile, which was determined by the assessment of the main health policy priorities, leading causes of mortality, hospital admissions, and the notification period (Table 2).

**Table 2:** Health Values According to the International Classification of Diseases (ICD-10) in Espírito Santo, Brazil, in 2011–2012.

Relevant diseases (ICD-10)	Political priorities	Mortality	Morbidity	Notification
**Infectious and parasitic diseases (A00-B99)**				
Tuberculosis (A15-A19)	**XX**			**XX**
Leprosy (A30)	**XX**			**XX**
Schistosomiasis (B65)	**XX**			**XX**
Dengue (A90)	**XX**			**XX**
Leishmaniasis (B65)	**X**			
Trachoma (A71)	**X**			
Adult AIDS (B20-B24)				**X**
Pertussis (A37)				**X**
Viral hepatitis (B15-B19)				**X**
Syphilis in pregnant women (A50)				**X**
Leptospirosis (A27)				**X**
Meningitis (A87)				**X**
Other bacterial diseases (A20-A64)			**X**	
Diarrhoea/gastroenteritis* (A09)			**X**	
Other diseases and intestinal infections (A01-A08)			**X**	
Cardiocirculatory diseases (I00-I99)	**X**			
**Myocardial infarction (I21-I22), stroke (I64)**				
Ischaemic heart disease (I20-I25)	**XX**	**XX**		
Cerebrovascular diseases (I60-I69)	**XX**	**XX**		
Hypertensive diseases (I10-I15)		**X**		
Other heart diseases (I26-I52)		**X**		
Varicose veins of the lower extremities (I83)			**X**	
Heart failure (I50)			**X**	
Other ischaemic heart diseases (I24)			**X**	
**Respiratory diseases (J00-J99)**	**X**			
Pneumonia (J10-J18)		**XX**	**XX**	
Chronic diseases of the lower airways (J40-J47)		**X**		
Neoplasms (C50, C53, C61)**	**X**			
Trachea, bronchi, and lungs (C33-C34)	**XX**	**XX**		
Remaining neoplasms (C17; C23-C24; C26-C31; C37-C41; C44-C49; C51-C52; C57-C60; C62-C66; C68-C69; C73-C81; C88-C89; C96-C97)		**X**		
**Infant and maternal mortality**: ***	**X**			
**Diabetes mellitus (E10-E14)**		**X**		
**Other diseases of the digestive system (K00-K22; K28-K64; K66, K80, K82-K93)**		**X**		
**Cholelithiasis and cholecystitis (K80-K81)**			**X**	
**Other diseases of the urinary system (N30-N39)**			**X**	
**Inguinal hernia (K40)**			**X**	

Source: Adapted from Cavaca et al. (2015) [13].

*Origin of the presumed infection

**Breast, cervical, and prostate cancers.

***Certain conditions in the perinatal period (P00-P96); congenital malformations and chromosomal abnormalities (Q00-Q99); respiratory diseases (J00-J99); certain infectious and parasitic diseases (A00-B99).

**Legend:** XX Coincide in two categories. X Appear in a single category

Diseases considered relevant by the print media in Espírito Santo were those that reached the health value of the state (Table 2) and were among the most reported diseases in the analysed period (Table 1). Therefore, diseases such as dengue fever, acquired immune deficiency syndrome (AIDS), hepatitis, diabetes, and malignant tumours were extensively covered during the analysed period, corroborating the tradition of media coverage of these disorders (Table 3).

**Table 3:** Priority Diseases (Health Value) Covered in the Newspapers *A Tribuna* and *A Gazeta* According to the International Classification of Diseases (ICD-10) in Espírito Santo, Brazil, in 2011–2012.

DISEASE (ICD-10)	NEWS	DEATHS	DEATH-RELATED NEWS INDEX	HOSPITALIZATIONS	HOSPITALIZATION-RELATED NEWS INDEX	NOTIFICATIONS	NOTIFICATION-RELATED NEWS INDEX
**Diabetes (E10-E14)**	433	2,125	0.20	4,853	0.09	#	#
**Dengue (A90)**	350	34	10.29	4,410	0.08	43,970	<0.01
**Stroke (I64)** *	280	0.00*	0.00*	4,313	0.06	#	#
**Malignant breast neoplasms (C50)**	227	519	0.44	2,876	0.08	#	#
**Myocardial infarction (I21-I22)**	214	3,571	0.06	2,462	0.09	#	#
**Adult AIDS (B20-B24)**	198	526	0.38	820	0.24	1,242	0.15
**Hypertensive diseases (I10-I15)**	167	2,041	0.08	4,526	0.04	#	#
**Pneumonia (J10-J18)**	119	1,871	0.06	24,389	<0.01	#	#
Cardiocirculatory diseases (I00-I99)**	100	829	0.12	46,978	<0.01	#	#
**Malignant neoplasms of the prostate (C61)**	100	600	0.17	1,248	0.08	#	#
**Viral hepatitis (B15-B19)**	95	84	1.13	226	0.42	1,029	0.09
**Malignant neoplasms of the trachea, bronchi, and lungs (C33-C34)**	78	818	0.10	890	0.09	#	#

*SIM does not specify deaths from stroke (I64); it only classifies them using data from the chapter on cerebrovascular diseases (I60-I69).

**The cardiocirculatory diseases listed separately in this chapter (I00-I99) were excluded from the sum.

^#^ Diseases without mandatory reporting

The DNMs (Table 4) were quantitatively identified by evaluating the prioritized diseases (Table 2) that were not covered in the print media (Table 1). Emphasis was placed on poverty-related diseases, including tuberculosis, leprosy, schistosomiasis, leishmaniasis, and trachoma. Diseases with outbreaks in the period, including pertussis and meningitis, some cancers, respiratory diseases, ischaemic heart disease, cerebrovascular diseases, and other diseases with high hospitalization rates in the state (inguinal hernia, cholelithiasis and cholecystitis, other diseases of the urinary tract, diarrhoea, and varicose veins of the lower extremities, among others) were also given limited coverage by the media in Espírito Santo.

**Table 4:** Diseases Neglected by the Media: Priority Diseases (Health Value) with Limited Coverage in the Newspapers *A Tribuna* and *A Gazeta* According to the International Classification of Diseases (ICD-10) in Espírito Santo, Brazil, in 2011–2012.

DISEASE	NEWS	DEATHS	DEATH-RELATED NEWS INDEX	HOSPITALIZATIONS	HOSPITALIZATION-RELATED NEWS INDEX	NOTIFICATIONS	NOTIFICATION-RELATED NEWS INDEX
**Tuberculosis (A15-A19)**	42	137	0.31	471	0.09	2,903	0.01
**Leprosy (A30)**	14	6	2.33	391	0.04	1,958	0.01
**Schistosomiasis (B65)**	3	33	0.09	6	0.50	1,075	<0.01
**Leishmaniasis (B55)**	2	3	0.67	9	0.22	265	<0.01
**Trachoma (A71)**	1	0	-	0	0.00	#	#
**Pertussis (A37)**	29	5	5.80	171	0.17	1,045	0.03
**Syphilis in pregnant women (A50)** *	1	0	-	336	0.00	1,317	<0.01
**Leptospirosis (A27)**	13	20	0.65	94	0.14	530	0.02
**Meningitis (A87)**	52	48	1.08	106	0.49	524	0.10
Other bacterial diseases (A20-A64)**	210	241	0.87	11,500	0.02	#	#
**Diarrhoea/gastroenteritis (A09)**	34	124	0.27	5,864	0.01	#	#
**Other diseases and intestinal infections (A01-A08)**	63	11	5.73	5,173	0.01	#	#
**Ischaemic heart disease (I20-I25)**	1	4,363	0.00	7,104	<0.01	#	#
**Cerebrovascular diseases (I60-I69)**	4	4,085	0.00	7,272	<0.01	#	#
**Other heart diseases (I26-I52)**	385	1,588	0.24	994	0.39	#	#
**Varicose veins of the lower extremities (I83)**	22	0	-	11,967	<0.01	#	#
**Heart failure (I50)**	29	0	-	8,038	<0.01	#	#
**Other ischaemic heart disease (I24)**	0	-	-	7,104	0.00	#	#
Respiratory diseases (J00-J99)**	34	446	0.08	13,058	<0.01	#	#
**Chronic diseases of the lower airways (J40-J47)**	3	1,596	<0.01	9,502	<0.01	#	#
**Malignant neoplasm of the cervix (C53)**	50	199	0.25	1,254	0.04	#	#
**Other neoplasms**	178	985	0.18	10,034	0.02	#	#
**Certain conditions in the perinatal period (P00-P96)**	8	738	0.01	8,543	<0.01	#	#
**Congenital malformations and chromosomal abnormalities (Q00-Q99)**	12	462	0.03	2,683	<0.01	#	#
Certain infectious and parasitic diseases (A00-B99)**	14	134	0.10	34,748	<0.01	#	#
**Other diseases of the digestive system**	277	967	0.29	31,907	0.01	#	#
**Cholelithiasis and cholecystitis (K80-K81)**	6	69	0.09	9468	<0.01	#	#
**Other diseases of the urinary system (N30-N39)**	2	454	<0.01	850	<0.01	#	#
**Inguinal hernia (K40)**	2	0	-	5,301	<0.01	#	#

*ICD-10 A50 corresponds to congenital syphilis and was used as a strategy to identify cases of hospitalization due to syphilis in pregnant women. The Information System for Notifiable Diseases (Sistema de Informação de Agravos de Notificação—SINAN) classifies the cases of syphilis in pregnant women, and these cases were faithfully represented in this table.

**The diseases listed separately in these chapters were excluded from the sum.

^#^ Diseases without mandatory reporting

It is important to highlight some aspects of the following diseases: other bacterial diseases (210 articles, excluding the diseases listed separately), other heart diseases (385 articles), other neoplasms (178 articles), and other digestive diseases (277 articles). These disease groups represented sums of classification sets from other diseases and were presented in this manner in the SIM and, for the elaboration of a reliable death-related news index, they were indicated in the same manner in Table 4. For this reason, they present a high frequency of articles in combination; however, if considered in isolation, they had a small coverage frequency and were considered as DNM.

According to the perceptions of key journalists, the DNMs identified included rare diseases, such as amyotrophic lateral sclerosis (ALS), leishmaniasis, Down syndrome, and verminoses. Therefore, “*Rare diseases and diseases that affect minority groups […]*,” as indicated by the journalists, were addressed.

The reasons highlighted for media coverage (or not) of certain diseases included the political and economic interests of the newspapers, their editorial line of work, and the organizational routine of the newsrooms. The health/disease news covered included recent news with a negative slant, news with pessimistic character, and diseases that were most serious and those that affected and killed more people, as evidenced in the speech below:

*[…] "The parameter [to cover the news] is*: *is the news recent*? *Is the news appealing*? *Will it reach many people*?*"*

In addition, the alleged public interest was highlighted, considering the common sense of citizens (such as aesthetic issues in health) and the emotional appeal that certain diseases have:

*“In principle*, *news is that which interests most people; it has to cause an impact*, *right […] it needs to be of interest to the reader*, *especially if it’s health news*, *it has to contain information that will interest as many people as possible*.*"*

## Discussion

### Diseases, media coverage, and indices

It can be observed that contemporary news production is often less sensitive to certain requirements, such as those considered to be epidemiological priorities. Assuming the logic of the market of consumer attention [15], information is disclosed in the manner of the advertisement of commodities, i.e., by offering news and advertisements. Health-related problems are regarded as consumer products offered to potential customers [16]. In this context of growing interest devoted to media sources, health is largely addressed by the reporting of the extraordinary, disasters, denunciations, advertisements, technological and scientific innovations, diseases of celebrities, and prescriptions of beauty treatments or healthy lifestyles involving individual responsibility and the consumption of goods and services. In general, it is an approach with a marketing bias and one decontextualized from the political and social reality in which it operates and interferes [3]. The words of one journalist interviewed corroborate this affirmation: *“[…] We need to sell newspapers*. *Although we know the importance that is given to the news*, *of the importance of promoting and discussing it*, *we also need to sell newspapers*.*"*

Espirito Santo is a Brazilian state that has a small land area (46.096.925 km^2^) if compared to the vast majority of the others Brazilian states [17]. It has 78 municipalities with a predominantly urban population [18], and 15% of the population living in rural areas [19]. Despite being a state that has a reasonable health coverage, social and health problems persist, not unlike the rest of the country, such as streets and public lighting paving deficiency, presence of open sewers and garbage accumulated in public parks, mainly peripheries and economically disadvantaged areas [18], favoring the occurrence of neglected diseases.

Such a scenario underscores the need to discuss the role of the media in issues related to health and disease, and in this case, not only with respect to the news value of health demanded and desired by consumers of media products [20]. Added to such values are prosaic day-to-day health topics, which usually do not represent news scoops, although they are relevant to the disadvantaged strata of the population. These strata, by lacking a voice in the political scene, become elements that are symbolically depreciated and vulnerable to threats but of no interest to the media, with the exception of their unusual or tabloid characteristics [2]. In this sense, the media invisibility or neglect of certain health problems serves as a form of social obliteration [6] because what does not exist is therefore considered non-existent and, although ignored, may be accessible and observable [21].

Therefore, the most promising conditions for the discussion of the results displayed are the voids observed in the form of the decreased media coverage of morbid conditions considered priorities in Espírito Santo, as assessed by their health values. In fact, the indices proposed herein were adopted more as promoters of such evidence than as precise measurements of media omission. Therefore, the estimates presented herein are useful only for the complex portrayal of iniquities and to promote debates about the public usefulness of the media. Furthermore, it is impossible to estimate complex categories (negligence)—in which multiple factors incur—only by the evaluation of the quantitative criteria adopted and considering calculations based on arbitrary “cut points.” Nevertheless, these values and estimates indicate a grey area related to the cultural perceptions and unspoken omissions that often obscure segments of an unfair health reality. Therefore, we reiterate that such estimates serve only to identify new categories—DNMs—that constitute topics that cannot be exhausted considering the scope of this study.

The death-related news index and its derivations, the hospitalization-related news index and the notification-related news index, indicate discrepancies that were determined by the number of news stories for every death, hospitalization, or notification relevant to the diseases in question. These indices were initially proposed by Hans Rosling (Rosling H. Swine flu alert! News/Death ratio: 8176. Available at: http://www.gapminder.org/videos/swine-flu-alert-news-death-ratio-tuberculosis/. Accessed on February 18, 2015.) in the context of the coverage of tuberculosis and were discussed by Peter Allebeck (2010) [22] in relation to the media frenzy about the pandemic H1N1 influenza in 2009 and the gap between what is disclosed by the media, what is feared by the population, and what constitutes real public health threats.

The author shows that, in 13 days, there were 31 deaths caused by H1N1 worldwide, whereas tuberculosis killed 63,066 people in the same period of time. However, the media reported 253,442 news stories about swine flu but only 6,501 news stories about tuberculosis. This scenario resulted in a high death-related news index for H1N1 (8176), which contrasted with the modest index for tuberculosis (0.1). As corroborated by this study, Allebeck (2010) [22] indicates that the death-related news index does not objectively represent a validated index to measure inconsistencies in the media coverage but a representation that indicates discrepancies between reported and actual threats and problems. Furthermore, he states that the mortality rates of the diseases considered to be most serious (e.g., mental health and trachoma in Espírito Santo) are not high and suggests the development of other indices to indicate such discrepancies. Therefore, the development of a hospitalization-related news index and a notification-related news index is aimed at disclosing gaps and the differences in needs versus visibility while considering the social and epidemiological reality in Espírito Santo.

Traquina (2008) [23] defined events that are catastrophic, unusual, tragic, and capable of dramatization, personalization, and death itself, to have important news value in the process of the selection and development of news articles. Furthermore, a low mortality rate often reduces the impact of the media coverage and its political weight [24]. Therefore, in the opposite direction of our assumptions, our results indicate the presence of 12 epidemiologically relevant diseases in Espírito Santo that were effectively covered, including diabetes, dengue, circulatory diseases, neoplasms, pneumonia, AIDS, and viral hepatitis (Table 3). Dengue and hepatitis achieved a death-related news index higher than 1, indicating the release of more than one news article for each death. Nevertheless, it is clear that, with regard to hospitalization and notification, these diseases presented newsworthiness that fell short of their epidemiological importance. We observed a correlation between the high media coverage of dengue and its incidence, with coverage peaks often overlapping with increasing epidemics [25]. The importance assigned to the newsworthiness of dengue is corroborated by the death-related news index of 10.29 news articles for each death.

Viral hepatitis represents a significant public health problem in Brazil and overseas. In addition, its epidemiological pattern has changed, owing to improved hygiene and sanitation, vaccination against hepatitis B, and novel diagnostic techniques for hepatitis C. However, socioeconomic heterogeneity and inequities in access to healthcare and the incorporation of advanced technologies of diagnosis and treatment remain national problems [26]. Therefore, the wide media coverage identified in this study and the high number of notifications can indicate increased attention to the problem, both with regard to the publicization of the disease (i.e., public debate, health education, and health prevention) and the improvements in disease diagnosis and notification.

It is known that the mandatory reporting of diseases is essential to promote health surveillance and to encourage protective and preventive interventions [27]. Therefore, the media coverage of health conditions that demand increased notification may represent an important measure of public health surveillance and planning [28, 29].

Another case involves neoplasms—diseases of epidemiological relevance and great media visibility in recent years [20]—on which a large number of related news articles was published during the study period, as also reported in other studies [30, 31]. According to Romeyer (2014) [32], the higher the disease severity, the higher its media coverage. The author compared the media coverage rates of cancer, Alzheimer's disease, and respiratory allergies in the French media, concluding that cancer was the most covered disease, in contrast to allergies. Among the factors that influenced such differentiation in the French context, the fact that cancer has already affected thousands of public figures and artists created many “alert providers” for the disease. In addition, there is increased interest on the part of the media regarding new studies and health policies concerning neoplasms, in addition to scientific controversies with outstanding news value.

In contrast to this scenario, Alzheimer's disease represents a condition with a few controversies, including the fatality inherent to the end of life, and respiratory allergies are addressed using repetitive discourse, surrounded by sparse news, often occupying the space of fillers in the news grid [32].

### Diseases, media silencing, and contradictions: Negligence

Among the diseases classified as neglected by the media (Table 4), tuberculosis, schistosomiasis, leprosy, ischaemic heart disease, and cerebrovascular disease are the most prominent because they contribute two relevance criteria to the health value in Espírito Santo (Table 2); even so, they do not achieve consistent newsworthiness. Yet, leprosy presented a death-related news index of 2.33, which indicates a good rate of media disclosure in relation to the number of deaths, perhaps not primarily because of its newsworthiness but rather because of its low mortality rate. However, similar to pertussis (5.80), meningitis (1.08), and other infectious and intestinal diseases (5.73) that are also expressed in these indicators as “media highlights” in relation to their mortality rates, with respect to notifications and/or hospitalizations, their newsworthiness lay below their epidemiological relevance, which is associated with poverty and access to SUS. Therefore, when analysing the media coverage of diseases, it is essential to consider the limitations (or even contradictions) of the representativeness of these indices.

Similarly, the limited coverage of ischaemic heart disease and cerebrovascular disease deserves attention because the news articles on the number of deaths due to heart attack and stroke were excluded (shown in Table 3). Therefore, although both groups of diseases cause many deaths, their low coverage may be related to either the lack of knowledge by journalists or the existence of little editorial interest to further clarify this group of diseases. Furthermore, although their ICD classifications are distinct, they are less popular than the designations "heart attack" and "stroke."

It is acknowledged that, while the media exposure of certain diseases fosters the development of social attention around them, media neglect contributes to the poor visibility and political sustainability of some diseases [6]. Therefore, the critique of the marketing bias in the approach to health and of the hegemonic newsworthiness criteria that guide the media coverage of diseases is based on the gap between the interests of the consumer attention market [15] and those of public health, particularly for the more vulnerable social sectors.

The evaluation of the agreement between the DNMs, considering the quantitative and qualitative results, indicated the acknowledgment of journalists only with respect to leishmaniasis and parasitic infectious diseases, such as verminoses. This finding points to the wide spectrum of diseases that affect some Brazilian regions and that are often unknown by the local press and characterize health inequalities.

In addition, other causes for concern are rare diseases and Down syndrome, which were not quantitatively covered in this study, as follows:

*"When the disease is very rare*, *we only put it in the paper if it is an interesting topic*. *Because there is no point talking about a new drug […] that will help only one percent of the population*.*"*

Nevertheless, these rare diseases are acknowledged, for example, by both personal motivations and the broad dissemination of strategic campaigns launched on social networks for ALS (*i*.*e*., the "ice bucket challenge"). In this campaign, individuals poured buckets of cold water on themselves to collect donations for charities involved in the treatment of motor neuron diseases, such as ALS; this campaign was endorsed by more than 17 million people in 2014:

*"[…] no one knew what this disease was* (ALS), *and then people came to know about it after the ice bucket story*.*"*

This example underscores the influence of media coverage of certain diseases on the public health agenda. The case of the “ice bucket challenge”, which resulted in more than 440 million views on social networks, collected USD 115 million in donations for the association that represents patients with ALS and other motor neuron diseases (ALS Association, United States) and tripled investments in research on ALS (BBC News. Available at: http://www.bbc.com/news/health-33640896. Accessed on October 26, 2015.), which is beginning to produce promising results [33].

We noticed in this study that the journalists interviewed have a limited and uncritical understanding of the reasons why media is being silenced about some public health ailments in ES, especially regarding Neglected Diseases. Somehow, when asked if they perceived some kind of media neglect, most of the respondents expressed difficulty in pointing DNMs, justifying this coverage through their professional *praxis*, considering it as a representation of everyday public interest. Thus, certain health issues are perceived as absent from the news only after a social alert makes them relevant by the media, as exemplified in the study with ALS and are not necessarily representative of the local epidemiological context. Therefore, the opinion of the journalists on the coverage or not of certain diseases and the nature of this coverage is often derived from private experiences, as seen in the account of one of the interviewees:

"*We met once a week*, *with several story suggestions*, *to decide what will and could be published or not*. *These suggestions come from*: *our own ideas (happens a lot with anyone who goes to the doctor and comes back with an agenda -laughs*!*); suggestions from colleagues*, *friends*, *family*, *which comes through questions*, *or personal experiences; we also keep track of other vehicles dealing with issues that have to do with us…* "(Journalist).

At present, the meeting agendas of the newspaper agencies act as filters that determine which issues will appear in the newspaper the next day [34]. Therefore, in most cases, the editorial lines and economic interests of these agencies guide such selections. The two main ES newspaper (*A Gazeta* and *A Tribuna*) kept a distinct and very clear editorial lines until the first semester of the 2000. *A Gazeta*, with a friendlier focus to the wealthier social class and *A Tribuna* with a more popular vocation. With greater access of class C, and the loss of readers to the electronic media, the newspaper *A Gazeta* chose to popularize and "rejuvenate", changing its standard format to tabloid. What happened was a decrease in sales of *A Gazeta* and an increase in *A Tribuna*, both in the number of readers and advertisers [35]. Currently readers belongs to a lower class, especially the ones reading *A Tribuna* which follows a national trend of increased intrusion of popular newspapers. Although they are more related to the lower classes, they concentrate their content on police news, football and urban problems [35].

Under the influence of economic interests, the two newspapers were always dependent and submissive to its biggest advertisers: state government, municipalities, large companies—*Vale*, *Aracruz / Fibria*, *CST / Arcelor Mittal Samarco*, *Garoto* and some trades retailers, such as supermarkets and appliance stores [35]. It is noteworthy that such interests little tangent the public health issues that are relevant for the disadvantaged populations.

Thus, the emotional and economic appeal of certain ills more direct health guidelines of ES than the epidemiological relevance itself. However, is the media coverage of catastrophic and extraordinary topics relevant to health? Or rather, what would be catastrophic and extraordinary **to** public health? The study results reveal the presence of DNMs with disquieting (catastrophic?) data to the public health in the state; yet, these DNMs are little valued by the media.

For example, leptospirosis (530 notifications and 20 deaths) is a zoonosis of great social and economic importance that is highly influenced by poor sanitary conditions and to which the entire population is susceptible. Pertussis (1045 notifications) and meningitis (524 notifications) are preventable and communicable diseases; therefore, the media can act assertively to publicize outbreaks, educate the population about signs and symptoms, and adopt specific measures for disease prevention. Schistosomiasis (1075 notifications) and leishmaniasis (265 notifications and also cited by respondents as important) are endemic in several municipalities in the state [12], which increases political concerns; even then, these diseases were covered by the newspapers only three and two times, respectively.

In addition to these examples, we could mention the diseases that are treated in primary healthcare services but that still have increasing numbers, including syphilis in pregnant women (1317 notifications), diarrhoea and gastroenteritis (5864 admissions), and uterine cancer (199 deaths), and even the verminoses mentioned by the interviewed journalists, among other rare conditions. In our view, these diseases represent dire, unacceptable, and highly relevant conditions to ensure media coverage and their inclusion in the public and political agenda.

In this context, problems begin to attract public/political attention when they reach a certain level of media visibility [36]. Therefore, as indicated by Hudacek et al. (2011) [37], this cycle involves the exposure or neglect of certain diseases in the public space, depending on the coverage or integration of these pathologies by the media, particularly when they have alert providers who perpetuate the cycle.

To better understand the exposure and negligence that permeate the media coverage of diseases, it is important to analyse the conformation of the journalistic field and the process of the publicity and media coverage of health.

### The journalistic field and the publicity of health

The journalistic field is a microcosm with its own laws that is defined by both its position in the global scenario and the attractions and repulsions of other microcosms [38]. Therefore, for an analysis of the media coverage of health issues, it is important to consider the practical rules that underlie the process of news production, the criteria of newsworthiness, and the productive routinization of editorial offices [38].

At the same time, it is also necessary to consider the routine of the journalistic professional as a definitive factor in understanding the way health topics are built and disseminated by the media, combined with a conscious problematization of the various economic, social, cultural, and political factors that this discussion addresses.

In the face of editorial constraints, routines, and thinking habits imposed without discussion, the media often produce a representation of the world formed by a philosophy of history as a meaningless succession of disasters and decontextualized facts, of which the system of relations where they are inserted (i.e., family structure, labour market, tax policy, etc.) is excluded [38]. In addition, there is a prevalent interest in unusual and immediate news stories that assume the role of “headlines,” to the detriment of actions that do not have immediate visible effects, thus promoting a fatalistic disengagement that is clearly favourable to the maintenance of the established order [38].

In addition, the coverage of diseases that are more well known by journalists prevails in the agendas because of the availability of relevant information and expert sources. This productive routine in newspaper agencies often precludes accurate surveys on diseases that are relevant and common but unknown (e.g., neglected diseases). Therefore, according to one respondent, some diseases have increased coverage because there are relevant active sources that are available to journalists:

*"[…] we have a gynaecologist who is our favourite and who speaks very well*, *and he is able to ask the patient to wait five minutes in the chair to talk to us*. *In some cases*, *he says*: *‘I cannot meet with you because this is a more severe case but I will talk to you in 15 minutes*.*’ So*, *these doctors end up having priority*.*"*

In addition, the media silencing about ailments related to poverty is given by a convergence of factors such as the fact that neglected diseases, in most cases, do not represent epidemiological emergencies [39] and they focus on inland regions and state peripheries [12], which becomes an obstacle for the social visibility and for the consequent media coverage. In addition to this there is the fact that, the little in-depth coverage of these social ills protects the local government from any responsibility for the identified iniquities [40]. Moreover, often the health department itself does not bother to disclose some health problems to the media or perpetuates a speech about absence of information relating to such diseases in an attempt to prevent alarmism of Public Health and conserve the state's political image.

For these reasons, it is extremely important to reiterate that health issues are part of a prerogative of public communication, communication of general interest [41], which presupposes a public space for publicity and debate [36], and the logic of journalistic production is only one of several other factors influencing the media coverage of health.

Therefore, the media coverage of health emerges from a series of discourses and competitive interests, far beyond the medical-health and media contexts. Political decisions, militant actions, influence sources, epidemics, the release of novel drugs, and technological and scientific discoveries act as alert providers and mobilize themselves to appear in the public scene [41]. Therefore, media coverage (and communication and health as a whole) constitutes an arena of power disputes.

According to Romeyer (2013) [36], it is possible to distinguish three characteristics of the health publicity process: the imposition of norms and behaviours via public discourse, debates of health challenges in the press, and the emergence of new social elements in search of legitimacy. With regard to the prescriptive bias, it is evident from the use of preventive messages that they are associated with the idea of risk, aimed at stimulating behaviours considered “normal” or in time to change a behaviour considered “risky.” In contrast, health news becomes prominent, particularly stories with distressing themes involving disasters or that reveal cases. The new social elements appear in the form of collective groups—forums, social networks, and associations—that often group together to make their voices heard and to influence public policies.

To better understand this process, health publicity should be analysed, considering its temporal and contextual relations (as part of political, institutional, and epidemiological priorities?). However, the protagonist of publicity is the effect of labelling/classification of the problem (in this case, the disease), which promotes its definition and public recognition. Moreover, publicity depends on the collective discourse, which can draw increased attention because of its alarmist approach and can contend the themes disclosed by the media and the actions/reactions of public authorities. Therefore, there is publicity in the passage and relationship between these elements, in a game of successive integration and reintegration of public issues [36].

Diseases can be labelled or classified in several ways, including through the diagnosis of a certain patient or an epidemic in a certain population. According to Rosenberg (2002) [42], diagnosis can classify, determine, and predict and, in doing so, it helps to establish and legitimize the reality that it identifies. According to the author, although diagnosis has been important in the history of clinical medicine, it has become particularly relevant in the late twentieth century, with the proliferation of cytological, chemical, and imaging examinations. Therefore, medical technology has facilitated the diagnostic process and has further promoted the understanding of disease as a whole.

Such a central organization of diseases into categories has also been used because of the bureaucratic requirements of hospital administrations and the plurality of contexts, from life and health insurance to discussions on epidemiology and public health policies. Therefore, it is possible to infer that disease classification has several effects. In this study, we observed the effects of disease visibility through their labelling/classification according to the ICD-10, the tenth International Statistical Classification of Diseases and Health-related Problems published by the World Health Organization (WHO), which aimed at standardizing the classification of diseases and health problems and in which a single category is assigned to each state/disease (ICD-10 code) (http://www.datasus.gov.br/cid10/V2008/apresent.htm).

In this cycle of visibility and public attention, the ICD-10 allowed the labelling, comparison of the information available in official databases, and identification of pathologies to which unequal weights are assigned during their coverage by the media. Diseases such as tuberculosis, schistosomiasis, leishmaniasis, trachoma, gastroenteritis, and inguinal hernia were neglected by the media in Espírito Santo. This classification by relevance according to the health value and the classification of the degree of neglect by the media served to call attention to the social importance of these diseases and the inequities perpetuated through their neglect.

### Fallibility, biopolitics, and future perspectives

However, we also question whether the evaluation of the newsworthiness—the proposal of the study—would not be an arbitrary biopolitical expression by the “input of phenomena peculiar to human life in the order of knowledge and power, in the sphere of political techniques” [43]. Rose (2007) [44] states that biopolitical strategies are not limited to matters of state and involve several strategies for the treatment of vitality, morbidity, and mortality at a desirable level. Therefore, would our strictly quantitative media propositions not be a political attempt to push the citizens toward essentially subjective definitions of the intervention spectra of human vitality?

In this instance, the assumption of our fallibility is revealed. Considering the difficulty of measuring subjective needs and epistemic (and empirical) setbacks of this phenomenological dimension of health, we assume that this combination of criteria for the construction of the health value would at least slightly address the most vulnerable problems (i.e., the fragility of the groups most affected socially) contemplated in policy priorities in Espírito Santo. However, the same priority is established using essentially epidemiological criteria and rarely (never?) considering the subjectivity of the health needs of the population. Considering these findings, other studies should be conducted to identify possible strategies to overcome this gap in the media coverage of DNMs [34].

Finally, it is known that agendas related to health and disease are mobilized by different interests, lobbies, and alert providers. Some diseases, such as those identified in this study, have high media coverage for various reasons, including the aggregation of a symbolic value derived from these different interests. Other diseases, known as DNMs, are neglected because they do not reach a high enough level of newsworthiness and, in most cases, they affect only ignored segments of the population. To give visibility to certain topics, it is essential to outline and develop a topic recognized as a political issue that is important enough for its consideration by public authorities.

In short, the high and low newsworthiness indicators proposed herein should also be considered to be symbolic representations pursuing other interpretations that transcend their mere incorporation into the literal and concrete. DNMs should also be considered in their symbolic dimension to be rhetorical figures that establish a political position that is wider than the quantitative dimension that they target and to which they are confined. Although similar to other epidemiological constructions, the rhetoric of this representation is based on the invisibility and omissions that are more symbolic than concrete and that demand a political position that, so far, has been little regarded in the academic domain.
